# Label-Free Biosensor Detection of Endocrine Disrupting Compounds Using Engineered Estrogen Receptors

**DOI:** 10.3390/bios8010001

**Published:** 2017-12-22

**Authors:** Rita La Spina, Valentina E. V. Ferrero, Venera Aiello, Mattia Pedotti, Luca Varani, Teresa Lettieri, Luigi Calzolai, Willem Haasnoot, Pascal Colpo

**Affiliations:** 1 European Commission–DG Joint Research Centre, Directorate Health Consumer and Reference Materials, 21027 Ispra, Italy; Rita.LA-SPINA@ec.europa.eu (R.L.S.); vaiello79@gmail.com (V.A.); luigi.calzolai@ec.europa.eu (L.C.); 2 European Commission–DG Joint Research Centre, Directorate Sustainable Resources, 21027 Ispra, Italy; valentina.ferrero81@gmail.com (V.E.V.F.); teresa.lettieri@ec.europa.eu (T.L.); 3 Institute for Research in Biomedicine, Università della Svizzera italiana (USI), 6500 Bellinzona, Switzerland; mattia.pedotti@irb.usi.ch (M.P.); luca.varani@irb.usi.ch (L.V.); 4 Authenticity & Bioassays, RIKILT Wageningen University & Research, Wageningen University, 6708 WB Wageningen, The Netherlands; willem.haasnoot@wur.nl

**Keywords:** estrogen receptor, 17β-estradiol, label-free assay, surface plasmon resonance, amplification, water

## Abstract

Endocrine Disrupting Compounds (EDCs) are chemical substances shown to interfere with endogenous hormones affecting the endocrine, immune and nervous systems of mammals. EDCs are the causative agents of diseases including reproductive disorders and cancers. This highlights the urgency to develop fast and sensitive methods to detect EDCs, which are detrimental even at very low concentrations. In this work, we propose a label-free surface plasmon resonance (SPR) biosensor method to detect specific EDCs (17 β-estradiol (E2), ethinyl-estradiol, 4-nonylphenol, tamoxifen) through their binding to estrogen receptor alpha (ERα). We show that the use of rationally designed ERα (as bio-recognition element) in combination with conformation-sensitive peptides (as amplification agent, resulting in increased responses) enables the detection of low parts per billion (ppb) levels of E2. As a proof of concept, this bioassay was used to detect E2 in (spiked) real water samples from fish farms, rivers and the sea at low ppb levels after concentration by solid phase extraction. In addition, the present SPR assay that combines a conformation-sensitive peptide with an array of ERα mutants is very promising for the assessment of the risk of potential estrogenic activity for chemical substances.

## 1. Introduction

Growing evidence in the last decades suggested that numerous chemicals, both natural and man-made, may interfere with the endocrine system and produce adverse effects in animals and humans. Scientists often refer to these chemicals as Endocrine Disrupting Compounds (EDCs).

These chemicals are found in many commercial products, such as plastic bottles, containers, metal food cans, detergents, flame-retardants, food, toys, cosmetics, drugs and pesticides.

17β-Estradiol (E2) and other natural hormones, natural chemicals, man-made chemicals and pharmaceutical products [1], are considered as EDCs because of their ability to mimic the effect of endogenous hormones [2]. They can interfere with the endocrine, immune and nervous systems of mammals [3]. Many of these EDCs have been found in the environments at very low concentration [4], but chronic low-level exposure might cause adverse biological effects in animals and humans [5]. For this reason, the REACH regulation (Registration, Evaluation, Authorisation and Restriction of Chemicals) imposes high level of protection for human health and the environment to the manufacturers of chemicals in the EU. To address these issues, simple, fast, accurate and sensitive methods for screening these compounds are required [6]. The most sensitive methods currently available for the detection of (anti)estrogenic activity are cell-based assays such as ER-CALUX, MELN, T47D-KBLuc and the Yeast Estrogen Screen (YES) assays [7]. Although these methods have been successfully developed to overcome animal testing and reach limit of detection of pg-ng/L, they have the disadvantages to require 24 h for test completion and specific laboratories equipment and personnel. In contrast, several bioanalytical techniques have been developed based on optical and electrochemical detection, in which the bio-recognition elements are usually antibodies, enzymes, receptors, nucleic acid, or whole cells [8]. Label free, antibody-based immuno-sensors have been used for environmental detection of EDCs [9,10,11,12,13], but the cost and difficulties to produce specific antibodies to different EDCs remains a limitation.

Interesting approaches using estrogen receptor proteins (ER) as a biorecognition element have been proposed as alternative methods to the use of antibodies [10]. ERα is a member of the superfamily of nuclear receptors [14,15,16] activating signaling pathways that regulate biological processes such as reproduction, embryonic development and homeostasis [17,18,19,20]. ERα is composed by five domains, three of which are well-structured functional domains: a modulating domain, a DNA-binding domain (DBD) and a ligand binding domain (LBD) [21,22,23]. Binding of ligands to the LBD of the wild type estrogen receptor (wt-ERα^LBD^) induces conformational changes, which in turn affect and regulate the hormonal pathways. In the presence of agonist ligands the so-called helix 12, which lies over the ligand-binding cavity, changes position in the presence of agonist or antagonist ligands. Different positioning of helix 12 and associated changes in other helices of wt-ERα result in different co-regulator binding surfaces and hence underlie the different physiological effects of agonists and antagonists compounds [24,25,26,27]. Rationally designed mutants in the ERα^LBD^ were produced to increase the affinity towards specific agonist/antagonist compounds [28] by replacing residue methionine (M) 421 with isoleucine (I) or phenylalanine (F). In comparison to wt-ERα^LBD^, M421F-ERα^LBD^ exhibits stronger binding affinity for E2, ethinyl-estradiol (EE2), and 4-nonylphenol (4-NP) whereas M421I-ERα^LBD^ has lower binding affinity for all investigated compounds compared to wt-ERα^LBD^ [28].

wt-ERα has been used to detect agonist/antagonist compounds in in vitro tests based on bioaffinity mass spectroscopy [29] and surface plasmon resonance (SPR) approaches [30,31]. However, the limits of detection are still orders of magnitude higher than those of cell culture bioassays. The advantage of using bioanalytical tools is their direct applicability for continuous assessment of EDCs [32]. SPR has been shown to be a versatile technique for biosensor applications [33] and novel and portable SPR devices are under development [34]. In addition, direct detection of agonist/antagonist compounds using label-free biosensors is challenging because their low molecular weights (typically <500 Da) are close to the inherent limit of detection of most label-free techniques [31]. 

An interesting method to bypass this problem is to exploit the ability of ER to change conformation upon ligand binding. Indeed, αβ/I peptide, a specific peptide motif belonging to ER interacting proteins, was demonstrated to recognize the active conformation of wt-ERα^LBD^ [35,36,37]. 

Here we propose an SPR based label free method that exploits both the ERα^LBD^ and a peptide (αβ/I) able to recognize the active conformation of agonist ligand bound ERα^LBD^. Different compounds, such as E2, can act as ER agonist molecules, promoting an ER active conformation that is specifically recognized by the αβ/I peptide. By contrast, binding of antagonist compounds such as tamoxifen causes conformational changes that prevent ER from binding the αβ/I peptide. In our proposed method, the specific binding of ligands is monitored through the ER interaction with the αβ/I peptide immobilized on an SPR sensor surface and used as recognition and amplification element. The assay is performed by incubating ERα^LBD^ with E2 and we show that the SPR binding response is directly related to the concentration of ligand bound to ERα^LBD^. Moreover, screening of an array of engineered estrogen receptors (wt-ERα^LBD^, M421F-ERα^LBD^, M421I-ERα^LBD^ and Y537S-ERα^LBD^) with a set of agonist and antagonist ligands shows that the assay is a fast method to detect the estrogenic activities of E2, EE2, 4-NP and tamoxifen. As a proof of concept, the wt-ERα^LBD^-based assay was applied to the detection of estrogen activity in water samples; the procedure and experimental conditions were adapted to create a robust and reproducible assay with great promise for future applications.

## 2. Materials and Methods

### 2.1. Materials and Reagents

Endocrine disrupting compounds (EDCs) such as 17β-estradiol (E2), ethinyl-estradiol(EE2), 4-nonylphenol (4-NP) and 4-OH tamoxifen (TAM) were purchased from Sigma-Aldrich (St. Louis, MO, USA). Stock solutions of all ligands were prepared in methanol; for SPR measurements, the stock solutions were further diluted with methanol into a series of concentrations, and then added to the final working samples in order to obtain the right ligand concentration and a final methanol percentage of 2%. Biotinylated-Dioxa (AEEA) and amine terminated peptides αβ/I (SSNHQSSRLIELLSR) are synthesized by Primm Srl (Milan, Italy). All the other reagents were acquired from general laboratory suppliers with analytical purity. Solutions were prepared in deionized water from water purification system MMilli-Q^®^ Integral (Millipore-Merck, Burlington, MA, USA). 

### 2.2. ERα^LBD^ Production and Purification

Wild-type ERα^LBD^ and its mutants were expressed in high yield in *E. coli* Rosetta 2(DE3)pLysS cells with a pET21 vector (Novagen) and then purified as previously described [28].

Single point mutants were generated by site-directed mutagenesis of the vector encoding the wild-type sequence using the QuickChange site-directed mutagenesis kit (Stratagene). The introduction of the desired mutations was confirmed by DNA sequencing. The PCR products are transformed into XL10-Blue supercompetent *E. coli* cells (Stratagene) and the plasmids purified according to standard techniques and transferred in *E. coli* Rosetta 2(DE3)pLysS cells for protein production.

### 2.3. Design of the Binding Assay

**Figure 1:**
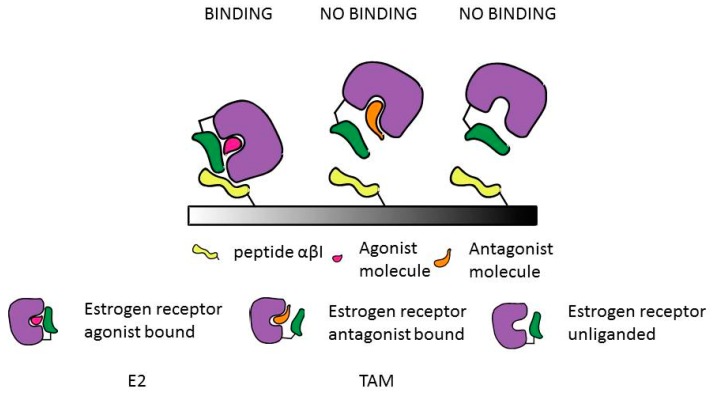
Schematic representation of the approach used for the detection of ERα^LBD^ agonist and antagonist compounds.

The SPR label-free binding assay is based on the ability of αβ/I peptide to recognize a ligand bound conformation of ER (also called active conformation upon agonist binding). Figure 1 is a schematic representation of the assay: αβ/I biotinylated peptide (peptide in yellow) able to recognize the active conformation of the ERα^LBD^ (violet) was directly immobilized on a neutravidin coated surface. After immobilization of the αβ/I, pre-incubated ERα^LBD^-ligand complex solutions were flowed over the peptide functionalized surface and the binding event of ERα^LBD^-ligand onto αβ/I peptide was monitored. In case an agonist molecule (magenta) is bound to ERα^LBD^, helix 12 (green) moves creating a conformation that is recognized by the αβ/I peptide. When ERα^LBD^ is free (un-liganded) or antagonist bound (orange), helix 12 might create a conformation that is not recognized by the αβ/I peptide. 

Monitoring the amount of ERα^LBD^-E2 complex bound onto αβ/I peptide is a direct detection of E2, because the amount of detected ERα^LBD^-E2 is proportional to the E2 concentration. The advantage of this assay format is that it is based on the recognition of a large molecule (the ERα^LBD^-E2 complex is around 29 kDa), which circumvents the issue related to the detection of small molecules (for instance E2 is 273.82 Da) with low responses in SPR-based detection methods.

### 2.4. SPR Measurements

#### 2.4.1. ProteOn XPR36™

All the binding experiments were performed with a ProteOn XPR36™ SPR instrument (BioRad), at 25 °C using a neutravidin modified chip (NLC sensor chip, BioRad, Hercules, CA, USA). With this instrument a 6 × 6 interaction array for the simultaneous analysis of thirty-six different experimental conditions was generated. NLC is a sensor chip with a neutravidin protein immobilized into a matrix of alginate polymer. The further coupling on the chip exploits the specific binding between neutravidin and biotinylated molecules. The chip was activated according to the manufacturer’s instructions by sequentially injecting 1 M NaCl and 50 mM NaOH for 60 s at 30 µL/min. The immobilization of biotinylated peptide (αβ/I) on the NLC chip surface was done in 20 mM Tris-HCl, pH 8, 20 mM NaCl (buffer A) for 300 s at 30 µL/min. The subsequent injection of ERα^LBD^-ligand complex was done in 100 mM potassium phosphate buffer, pH 7.2, 20 mM NaCl, 2% methanol (buffer B) for 240 s at 100 µL/min. The biotinylated αβ/I peptide was immobilized in vertical way (left panel, Figure S1) and the peptide surface was, then, washed with buffer B for one hour. ERα^LBD^-ligand complexes were then flowed over the αβ/I peptide in horizontal way (right panel, Figure S1), and its binding was monitored. 

For each binding measurement, triplicate independent solutions were prepared for receptor-ligand mixture under investigation. Error was calculated using triplicates.

To assess the amount of immobilized peptide leading to an optimal SPR response, five different concentrations of peptides (0.1, 0.6, 1.4, 2.2 and 17 µg/mL) were injected in 5 channels (Figure S1, vertical way), and one channel was used as reference with buffer A.

To determine the best peptide concentration for ERα^LBD^-ligand binding, a mixture of ERα^LBD^ (4 µg/mL, 134 nM) and E2 (405 ng/mL, 1.5 µM) was prepared and flowed over the different peptide concentrations. Buffer B and free ERα^LBD^ were injected at the same time as controls. 

To determine the limit of quantification of our assay, mixtures of wt-ERα^LBD^ (134 nM) were prepared and incubated with different E2 concentrations (0.03, 0.3, 0.8, 2.7, 4, 8, 12, 27 ng/mL and 405 ng/mL). Buffer solution and wt-ERα^LBD^ without ligand were prepared and injected at the same time as controls. After each measurement, the peptide functionalized surface was regenerated by injecting 10 mM H_3_PO_4_ for 20 s at 100 µL/min in both flow directions to remove the ERα^LBD^-ligand complex from the modified functionalized peptide surface. Triplicate measurements were performed for each receptor-ligand concentration under investigation. 

To test the specific interaction of the estrogen receptors towards the αβ/I peptide, the same experiments of binding were performed with transthyretin protein (TTR), which is not supposed to bind the peptide. TTR at 134 nM, without and with E2 at 1.5 µM was injected onto the peptide coated surface and the SPR signal monitored. 

Furthermore, experiments were performed to determine the affinity of the αβ/I peptide to the wt-ERα^LBD^-E2 complex. Serial dilutions of wt-ERα^LBD^-E2 were prepared starting from wt-ERα^LBD^ 134 nM and E2 200 nM, then diluting 1:2 for five times. The wt-ERα^LBD^-E2 complex solutions were then flushed over the peptide coated surface and each binding curve is fitted to Langmuir isotherm.

To screen the binding abilities of ERα^LBD^ mutants bound to selected ligands, receptors were pre-incubated and then flowed over the peptide surface. Respectively 134 nM wt-ERα^LBD^, M421F-ERα^LBD^, M421I-ERα^LBD^ and Y537S-ERα^LBD^ were injected as free receptor, or incubated with 1.5 µM E2, EE2, 4-NP and TAM. The reference solutions were prepared by adding 2% methanol to buffer B alone and to ERα^LBD^ solutions without ligand. ERα^LBD^-ligand, ERα^LBD^-methanol and the buffer-methanol mixtures were incubated for 1 h at 4 °C. Triplicate solutions were prepared for each receptor-ligand mixture under investigation. 

#### 2.4.2. Biacore 3000

The binding experiments and water sample analyses were performed with a Biacore 3000 and carried out by immobilization of the amine-peptide directly to a CM5 chip. The amine terminated αβ/I peptide was dissolved in acetate-buffer (pH 4.5) in a concentration of 100 µg/mL and injected over an EDC/NHS activated CM5 chip surface and a response of 800 RU was recorded. 

The final optimized assay conditions were as follows: 100 μL of the wt-ERα^LBD^ (60 nM) in HBS-EP (10 mM Hepes, 150 mM NaCl, 3 mM EDTA, 0.005% Tween-20) buffer was mixed in the Biacore with 100 μL of E2 standard/sample in 20% methanol and of this mixture 90 μL was injected at 30 μL/min with HBS-EP as running buffer which was followed by the injection of 10 mM NaOH for 0.5 min for the regeneration. For calibration, E2 concentrations of 0.63, 1.25, 2.5, 5, 10 and 20 ng/mL were used. This bioassay was then used to test (spiked) Milli Q (MQ) water, tap drinking water, tap process water, real field water samples from fish tanks coming from the UK and river and sea water samples coming from Slovenia. The water samples were spiked with different concentrations of E2 (10, 20 or 50 ng/L (ppt)) and concentrated with by Solid Phase Extraction (SPE) for 200 times prior to SPR assay. The final sample extract contained 20% of methanol.

Furthermore, the real field water samples were analysed with the bioassay and the results compared with GC-MS (Gas chromatography-mass spectrometry) data. 

### 2.5. Water Sample Preparation by SPE

Field water samples were kept frozen until the day before being analysed. The thawed water sample was filtered through a folder paper filter (S&S 595 ½) with a pore size of 4–7 μm) followed by an Acrodisc syringe filter of 0.45 μm (Pall) to avoid clogging of the SPE column (as was experienced before). Sample volumes of 200 or 500 mL of the filtered water were brought to pH 5.2 by the addition of 5 mL of acetate buffer (pH 5.2). Hereafter, 50 µL of Helix Pomatia was added and the whole left overnight at 37 °C (mild shaking in a water bath). The column (Bond Elut C18 3 cc/500 mg of Varian (1210–2028) was activated by passing through 2.5 mL of methanol followed by 2.5 mL sodium acetate buffer (pH 4.8). The water sample was passed through the column, and thereafter the column was washed with 1.5 mL of sodium acetate buffer, 3 mL of water, 1.5 mL sodium carbonate buffer, 3 mL water and 2 mL methanol/water (50/50; *v*/*v*). The column was dried and eluted with 4 mL of acetonitrile. After overnight storage, the eluates contained solids and they were removed by filtration (Whatman no. 1), the filter was washed with acetonitrile and it was evaporated and dissolved in 200 μL of methanol. This was divided in 4 portions of 50 μL, representing 50 mL of water sample each, and stored in closed vials at 4–6 °C until analysed by the Biacore 3000.

### 2.6. GC-MS Analysis

The multi residue method (RIKILT SOP 1160) was developed for the detection of 27 anabolic compounds (including E2 and EE2) from 0–1 µg/L. Of the methanol extracts of the water samples, 25 µL portions (equivalent to 25 mL of water) were evaporated and 25 μL of the derivatization reagent *N*-methyl-*N*-trimethyl silyltrifluoroacetamide (MSTFA) was added. The vials were vortexed and the reaction mixture was incubated during 1 h at 60 °C. After incubation, the reaction mixture was evaporated to dryness under a stream of nitrogen at 55 °C. The residue was dissolved in 50 μL of isooctane and transferred into a glass injection insert and transferred into the automatic injector (Varian, type CP-8400, Varian, Inc, Walnut Creek, CA, USA) of the GC-MS-MS (Varian type CP-3800 and type 1200 L, (Varian, Inc, Walnut Creek, CA, USA) and 2 μL were injected into the GC-column (VF-17MS, 30 m × 0.25 mm ID, film thickness 0.15 μm (Varian CP8981, Varian, Inc, Walnut Creek, CA, USA).

## 3. Results and Discussion

### 3.1. Optimisation of the Assay Parameters

Optimization of the procedure for peptide coating of the SPR surface and ERα^LBD^ concentration was performed on the ProteOn XPR36™ (BioRad, Hercules, CA, USA). Biotinylated αβ/I peptides were immobilized on a neutravidin modified sensor chip to form a monolayer through the biotin-neutravidin specific interaction. To determine the amount of immobilized peptide leading to an optimal SPR response, five different αβ/I peptide concentrations (0.06, 0.25, 0.6, 1.0 and 7.5 μM) were flowed over the sensor surface (Figure S2. SI, Panel A). Buffer A without peptide was used in the reference channel (Figure S2. SI, Panel A, black continuous line). The amount of αβ/I peptide bound to neutravidin on the surface was determined and the corresponding SPR response plotted versus the peptide concentration in Figure S2. (SI, Panel B). A maximum of 650 SPR response units (RU) was determined for the peptide immobilization, with a signal variation of 8%. In addition, the binding response reached a plateau for peptide concentrations higher than 1 μm.

For the optimization of the assay, E2 was chosen as model-ligand due to its high affinity towards the ERα^LBD^ binding pocket. Indeed, E2 generates the active conformation of ERα^LBD^ (agonist bound) specifically recognized by the αβ/I peptide. Preliminary tests with a range of ERα^LBD^ concentrations from 10 to 200 nM were performed to determine the optimal amount of receptor to be used for the following experiments (data not shown).

wt-ERα^LBD^ concentration of 134 nM was found to provide a high response signal and used for the subsequent experiments. ERα^LBD^ and E2 at the saturating concentration of 1.5 μM were pre-incubated for 45 min at 4 °C and then injected on SPR channels functionalized with different amounts of αβ/I peptide (Figure S3. SI). 

The signal resulting from the binding of the wt-ERα^LBD^-E2 complex on the peptide-coated chip increases with the amount of immobilized peptide (Figure S3. SI, Panel A). A signal plateau around 1100 RU, corresponding to the saturation of wt-ERα^LBD^-E2 complex on the surface, was found at peptide concentration above 0.6 μM (Figure S3. SI, Panel B), which was selected for subsequent experiments since it provides satisfactory signal to noise ratio and a good coverage of the surface. Furthermore, the affinity of the αβ/I peptide to the wt-ERα^LBD^-E2 complex was measured as described in materials and methods. An apparent average K_D_ value of 2.5 × 10^−9^ ± 0.7 × 10^−9^ M was determined.

The specificity of the interaction of the estrogen receptors with the αβ/I peptide in our assay was verified by replacing ER with an unrelated protein with similar surface charge, TTR.

No SPR response was detected when 134 nM TTR, without and with E2 at 1.5 µM, was injected over the αβ/I peptide coated surface (Figure S4. SI).

Reproducibility of the SPR measurements and regeneration of the chip surface were also tested. After binding of wt-ERα^LBD^-E2 complex to the immobilized αβ/I peptide (Figure S5. SI, Step 1), the ER was removed by washing the SPR chip with 10 mM H_3_PO_4_ at 30 μL/min for 18 s (contact time with the chip) [38], regenerating the peptide surface for further testing (Figure S5. SI, Regeneration 1). After regeneration, the wt-ERα^LBD^-E2 complex was flowed over the peptide surface as before. The procedure was repeated twice (Figure S5. SI, Step 2, Regeneration 2, Step 3). Repeated injections of the same wt-ERα^LBD^-E2 complex solution before and after regeneration gave similar SPR responses (Figure S5. SI) with reproducibility within 6% of variation. These experiments demonstrate that the peptide coated surface can be successfully regenerated. The ease and effectiveness of the regeneration steps and the reproducibility of the SPR response are a key asset for the assay. Furthermore, the test can be completed in significantly shorter time than that required by cell-based assays. Surface activation and peptide immobilization are performed in 20 min; injection of the wt-ERα^LBD^-E2 and determination of its binding response are achieved in only 4 min. After that, the SPR chip can be regenerated, washed and be ready for further ER injections and testing. 

### 3.2. Limit of Detection, Sensitivity and Specificity of the Assay for wtERα^LBD^ and E2

**Figure 2:**
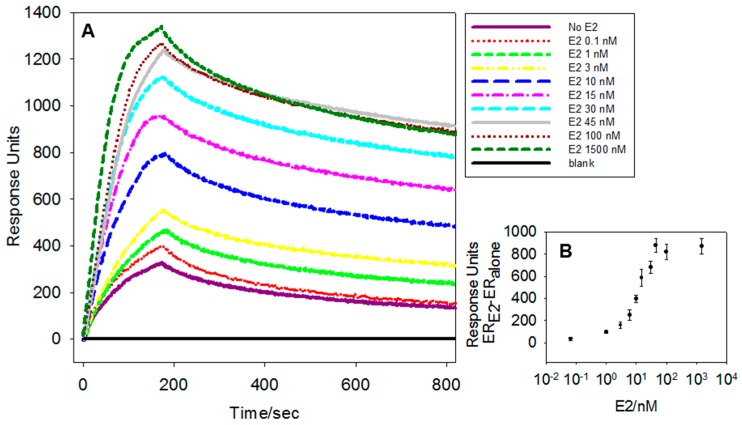
(**A**) Surface plasmon resonance (SPR) sensorgrams of the interaction between 134 nM wt-ERα^LBD^ with different E2 concentrations (0.1, 1, 3, 10, 15, 30, 45, 100, 1500 nM); (**B**) The SPR signal recorded 60 s after the injection and is plotted as a function of E2 concentration.

After optimization of the assay, the limit of detection (LoD) for the wtERα^LBD^-E2 system was determined by preparing mixtures of 134 nM wt-ERα^LBD^ with increasing amounts of E2, ranging from 0.1 to 1.5 µM. The wt-ERα^LBD^-E2 mixtures and the wt-ERα^LBD^ ligand free solutions were prepared in triplicate, incubated at 4 °C for one hour and injected over the sensor surface. E2 binding to wt-ERα^LBD^ generates an ER active conformation (agonist bound) specifically recognized by the αβ/I peptide. Binding of ERα^LBD^ to the immobilized peptide is expected to produce an SPR signal proportional to the amount of E2, since the ER concentration is kept constant in our assay. The SPR responses for different E2 concentrations are shown in Figure 2, Panel A. The SPR response recorded 60 s after the end of injection was used for quantification purposes. Increasing concentrations of E2 generate a proportional increase of signal response. Injection of ligand free wt-ERα^LBD^ results in a SPR response of 300 RU within 10% error, whereas wt-ERα^LBD^-E2 complex at 1.5 µM E2 shows a SPR response of 1200 RU within an error of 6%. The signal response can be fitted with a linear regression up to 30 nM with R^2^ of 0.9. A plot of the SPR response of wt-ERα^LBD^-E2 binding to the αβ/I peptide versus the E2 concentration is shown in Figure 2, Panel B.

The limit of detection (LoD) was defined as the E2 concentration producing an SPR response above the response of the ligand free wt-ERα^LBD^ (300 RU) plus 3 times the standard deviation (3 × 30 RU),corresponding to 1 nM or 0.20 ng/mL of E2. This LoD, although higher than that of LC-MS or radioimmunoassay based methods [39,40], this is equal or higher to the typical concentration of E2 found in contaminated real water samples. For instance, the concentration of E2 in the river Ahr at Blankenheim is in the order of 4–12 ng/L [41] and 122–631 ng/L in Rio de la Plata, Argentina [42]. However, the LoD could be further improved by pre-concentrating the sample through, for instance, the use of SPE columns or miniaturized pre-concentration column tools as the one developed by Heub S. et al. [43]. 

### 3.3. Testing Water Samples

**Figure 3:**
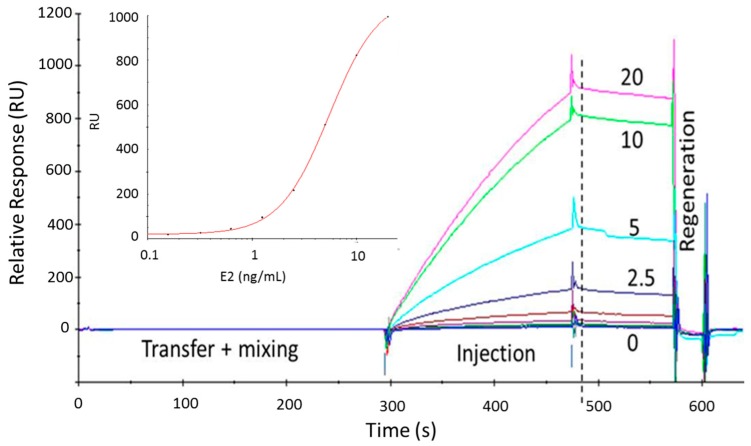
Referenced SPR sensorgrams obtained in the Biacore 3000 with E2 concentrations ranging from 0 to 20 ng/mL. The standard solutions in 20% methanol and the wt-ERα^LBD^ in HBS-EP (10 mM Hepes, 150 mM NaCl, 3 mM EDTA, 0.005% Tween-20) buffer were transferred and mixed in the Biacore (1:1; *v*/*v*). Of this mixture, 90 μL was injected over the peptide-coated sensor surface at a flow rate of 30 μL/min using HBS-EP as running buffer. After the injections, the responses were measured (dotted line) and used for the construction of the calibration curve (insert). The binding of the ER was regenerated by the injection of 10 mM NaOH for 0.5 min. The total time of each cycle was 11 min of which 5 min were used for the transfer and mixing.

For testing water samples, the bioassay was optimized with just one wt-ERα^LBD^ concentration (1 μg/mL) and one αβ/I peptide amount (800 RU) coated on the chip surface, to create the fastest, most robust and easiest to apply assay format suitable for routine testing. In this case, the amine terminated αβ/I peptide was immobilized over an EDC/NHS activated CM5 chip. For calibration, several solutions of wt-ERα^LBD^-E2 complex with different concentrations of E2 were prepared and injected over the αβ/I peptide. This coated biosensor chip could be used for more than 150 injections of samples/standards, and for weeks. The mixing (1:1; *v*/*v*) of the wt-ERα^LBD^ solution (60 nM (1 μg/mL in HBS-EP buffer)) with the standards and samples (in 20% methanol) was performed automatically in the Biacore 3000 and at room temperature creating similar incubation times between injections. No pre-incubation of the complex between wt-ERα^LBD^-E2 was applied because of the limited effect on the SPR responses. Therefore, the assay was carried out faster. In addition, the amount of methanol in the sample was varied between 0 and 20% and only a little signal reduction was observed with methanol >2% but a clear difference between the blank and positive samples was recorded. This result is very convenient because it allowed testing of the SPE eluate in methanol after less dilution (5 times only). Moreover, the use of HBS-EP buffer, as ERα dilution and running buffer showed larger differences between the blank and positive samples/standards in comparison to the phosphate buffer. Regarding the stability of ERα, it was found that diluted ERα could be stored during 1 week at 4–6 °C which is convenient for routine testing, whereas better regeneration was recorded by the10 mM NaOH for 0.5 min compared to 10 mM H_3_PO_4_ as applied in the ProteOn XPR36™. Overall, the assay time, including transferring and mixing of the ERα and the standards/samples and applying a 3 min injection at 30 μL/min and 1 min regeneration, was 11 min. These easy, fast and stable conditions made the test more suitable for routine testing. An example of an unreferenced (without the subtraction of the reference channel response) sensorgram is shown in Figure S6 and referenced sensorgrams and the calibration curve are shown in Figure 3 and the LoD was determined as 0.6 ng/mL. 

The optimized set-up was then used to test water samples with an expected level of EDCs in the order of low ppt (pg/mL). Since our assay works at the low ppb level (range of the calibration curve was from 0.5–10 ng/mL (Figure 3)), the water samples were concentrated up to 200 by SPE. In order to prove the robustness of the bioassay, several water samples (millipore water (MQ), drinking water, process water, water from a fish farm and river water samples) were tested with and without E2 spiking. MQ water sample was spiked with 0, 10, 20 and 50 ppt of E2, pre-concentrated by SPE from 50 to 0.25 mL (200 times), and the obtained responses (and recoveries) were 10, 80 (60%), 230 (64%) and 370 RU (37%), respectively. Although the recoveries varied between 37 and 64%, the 10 ppt E2 addition was clearly detectable and the limit of detection was estimated at <5 ppt.

The tap water samples (*n* = 2) and the fish farm water samples (Figure S2 were also analysed in the same way without and with the addition of 20 ppt of E2 and all spiked samples were clearly detected with responses from 120 to 270 RUs (compared with responses of 5 to 18 RUs for the blanks) and with recoveries varying from 38 to 75%. 

Real water samples (*n* = 8) from fish farms in the UK (*n* = 3), Slovenia (*n* = 2 (Bay of Piran)) and river water samples from Slovenia (*n* = 3), with and without the addition of 50 ppt of E2, were tested after SPE with a 200 times concentration. Prior to their analysis, these real samples were hydrolysed with Helix Pomatia overnight at 37 °C to convert possible conjugated estrogens (glucuronides and sulphates) into the free compounds.

Sensorgrams of one of the water samples from the UK (SP3), with and without the addition of E2, are shown in Figure S7 together with the responses (insert) obtained with all 8 samples and 2 standard (10 ng/mL) responses for comparison (10 ng/mL = 100% recovery). The non-spiked real water samples showed responses varying from 12 to 15 RU with an average of 14 ± 2 RU (corresponding with E2 concentrations in the samples of <5 ppt) and the spiked samples from 294 to 416 RU with an average of 374 ± 39 RU (corresponding with an average recovery of 74 ± 7%). As controls, Millipore Q (MiliQ) samples (*n* = 2) with and without the addition of 50 ppt of E2 were tested which resulted in comparable average responses (12 ± 0 and 386 ± 54 RU) and a comparable recovery (81%).

Additional water samples (*n* = 4) taken from a Slovenian fish farm, Slovenian rivers (*n* = 2) and the Gulf of Trieste were tested after a 500 times concentration by SPE and these resulted in an average response of 10 ± 6 RU which corresponds with a concentration of E2 of <1 ppt. The MQ controls (blank and spike at 10 ppt) gave responses of 15 and 469 RU with a recovery of 66%.

No positive water samples were found with the biosensor receptor test except for E2 spiked samples. In order to confirm the results, the same samples were tested by GCMS and no E2 and EE2 and other steroids were detected (<5 ppt) and the average recovery of the 50 ppt spiked samples (*n* = 8) was 76 ± 7%. The comparison of the data proves that wt-ERα^LBD^ bioassay is reliable and it can be successfully used to determine the amount of E2 in real water samples at low ppt levels.

### 3.4. Array of ERα Receptors Mutants

In this work, the SPR experiments have been performed to test the peptide-specific recognition of the conformation of complexes formed by wt-ERα^LBD^ and the mutants M421F-ERα^LBD^ and M421I-ERα^LBD^ with agonist and antagonist compounds [28]. The mutant Y537S-ERα^LBD^ has been used as positive control because of its permanent ligand-bound mode or active conformation, even in the absence of ligand. The permanent active conformation is due to the substitution of the Tyrosine into Serine amino-acids [44]. 

**Figure 4:**
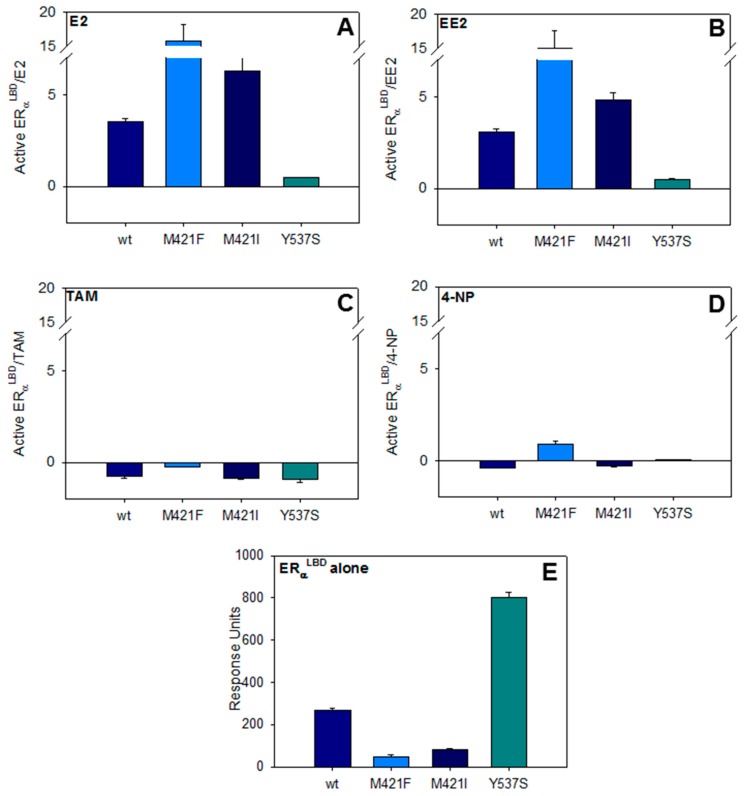
Comparative ratio of ERα^LBD^ ligands complexes towards the ligands (**A**) 17β-estradiol (E2); (**B**) ethinyl-estradiol (EE2); (**C**) 4-OH tamoxifen (TAM); (**D**) 4-nonylphenol (4-NP); and (**E**) ERs free binding response.

Ligand-free receptor was used as a control resulting in a limited amount of interactions with αβ/I peptides (Figure S8A–C, black line and Figure 4E). This unexpected binding is probably due to the flexible structure of ERα^LBD^, which may lead to partially active conformation [45]. Besides, the results also show that in the absence of ligand, αβ/I peptide is able to recognize the permanent active conformation of Y537S-ERα^LBD^ mutant (Figure 3D, black line).

After incubation of ERα^LBD^ receptors with 1.5 µM of E2, EE2, 4-NP or TAM, respectively, different ERα^LBD^–ligands complexes were flowed over the peptides coated chip and the sensogram is plotted in Figure S8.

In order to determine and compare the increase/decrease of SPR signal for the different ERα^LBD^ receptors and ligands, we define the parameter *R* as a ratio:

$$R=\frac{{\mathrm{Intensity}}_{{\mathrm{ER}\alpha }^{\mathrm{LBD}}-\mathrm{ligand}}-{\mathrm{Intensity}}_{\mathrm{free}-{\mathrm{ER}\alpha }^{\mathrm{LBD}}}}{{\mathrm{Intensity}}_{\mathrm{free}-{\mathrm{ER}\alpha }^{\mathrm{LBD}}}}$$

This parameter indicates the increasing (or decreasing) amount of ERα^LBD^ in the active conformation normalized to free ERα^LBD^. In Figure 4, the parameter *R* (Active ERα^LBD^/ligand) is plotted for each ERα^LBD^ mutant (wt-ERα^LBD^, M421F-ERα^LBD^, M421I-ERα^LBD^, Y537S-ERα^LBD^) and for the ligands E2, EE2, 4-NP and TAM.

In Figure 4, the *R* of each ligand (Active ERα^LBD^/ligand), incubated with ERα^LBD^ mutants and flowed over the αβ/I peptide, are presented. 

E2 and EE2, incubated with ERα^LBD^ mutants provoke a clear positive ratio *R* with a modulated response in the following order: Y537S-ERα^LBD^ < wt-ERα^LBD^ < M421I-ERα^LBD^ < M421F-ERα^LBD^ (Figure 4, Panels A and B). This trend is probably due to the higher affinity of M421F-ERα^LBD^ for E2 and EE2 [28] as compared to other receptor which results in a higher percentage of M421F-ERα^LBD^ in the active conformation, that is, higher recognition by αβ/I peptides. As expected, the ratio *R* resulting from the binding of Y537S-ERα^LBD^ complex formed with strong agonists to αβ/I peptide is only marginally increased because of its natural, active conformation even in the absence of agonist ligands as mentioned above [44].

The evidence of the Y537S-ERα^LBD^ mutant being in a high percentage in the active conformation is much clearer observing Figure 4E where it is possible to observe the different amount of the ligand-free mutants being in active conformation (Y537S-ERα^LBD^ > wt-ERα^LBD^ > M421I-ERα^LBD^ > M421F-ERα^LBD^).

The SPR responses are noticeably different for TAM (Figure 4, Panel C) where the normalized ratio (*R*) for all ERα^LBD^ mutants is found negative since the SPR signal for ERα^LBD^ -TAM complexes is lower than the reference (ligand free ERα^LBD^). This result confirms the interaction between TAM and the mutants binding pocket inducing a conformational change in an antagonist bound conformation, which is not recognized by αβ/I peptide. 

The weak agonist ligand 4-NP, generates a positive *R* for M421F-ERα^LBD^ mutant (Figure 4D, cyan) and negative *R* for the other mutants (Figure 4D). The lower affinity of 4-NP for ERα^LBD^ does not allow to establish a clear SPR response trend; however, the statistical relevance of the data is demonstrated by *t*-test. In details, the comparison of the SPR response for ligand free and 4-NP bound wt-ERα^LBD^, M421F-ERα^LBD^ and M421I-ERα^LBD^ are significantly different (p values calculated by *t*-test are 0.0002, 0.0002 and 0.0004, respectively) whereas for Y537S-ERα^LBD^ the *t*-test is not significantly different (*p* value 0.4154). From these analyses, it is tempting to say that the ‘positive /negative’ *R* pattern might indicate the presence of weak agonists in an unknown sample. 

These results suggest that the array of mutants can be used advantageously for a fast screening of the presence of agonist/antagonist compounds in an unknown sample. In specific, clear positive and negative *R* (*R* > 1 or *R* < 1) for the four receptors suggest the presence of an agonist or antagonist compounds, respectively. The combination of positive and negative *R* might indicate the presence of weak agonists. These different ratio pattern can thus give important information on the presence or not of potential estrogenic compounds in unknown samples that have to be nevertheless further identified in cellular environment.

To improve the identification capability of the compound by the present assay, more studies need to be carried out focusing on the determination of the relative ratio *R* of the studied mutants for more compounds at different concentrations. The large number of variable (concentration, EDCs compounds with associated affinity towards ERα^LBD^) need to be integrated by an algorithm analysis for further pattern recognition analysis. 

The present assay is complementary to conventional methods of determination of (anti)estrogenic activity traditionally performed by ER-CALUX, MELN, T47D-KBLuc and the Yeast Estrogen Screen (YES) assays. These tests are all cells-based bioassays requiring stably transfected transcriptional activation of responsive elements (luciferase for the three former assays and galactosidase for the last) [7].

Indeed, the SPR assay developed in this study allows discriminating the presence of an antagonist ligand from a situation where no ligand is present in a sample (see Figure 3). These two events are not distinguishable on cell-based assays, unless a co-injection of an agonist is carried out. Furthermore, the cell-based assay can be affected by different mechanisms, such as cytotoxicity and bioavailability of the compounds, while the SPR response is a direct measurement of binding of a compound. In addition, ERα^LBD^ assay is performed in 1 h rather than 24 h as required for a cell-based assay. Moreover, being a cell free assay, it does not need specific laboratory equipment and qualified personnel. Finally ERα^LBD^ assay can detect different compounds at the same time by using different ERα^LBD^ mutants and multiple channels (Figure S1). The advantage of using a cell-based bioassays is the higher sensitivity (20–40 pg/L for E2) [7] in comparison to ERα^LBD^ assay (~20 ng/L for E2); however, the sensitivity issue can be overcome by pre-concentrating the sample [43].

## 4. Conclusions

In conclusion, a SPR-based assay was designed for measuring the binding of agonist and antagonist compounds to ERα^LBD^. This assay is based on the immobilization of αβ/I biotinylated peptide, which specifically recognizes the conformational change of ERα^LBD^ upon binding to E2. This assay system allows easier detection of small molecules by SPR, since it is based on the ability of the αβ/I peptide to specifically recognize the ER_α_^LBD^-E2 complex, thus leading to a great change in SPR response. The limit of detection of the SPR assay is 0.60 ng/mL for wt-ERα^LBD^-E2 complex, which can be improved by pre-concentrating the sample. E2 spiked real water sample were successfully tested by wt-ERα^LBD^-E2 bioassay after SPE concentration and the results are in agreement with the GC MS data. Moreover, an array of engineered estrogen receptors (wt-ERα^LBD^, M421F-ERα^LBD^, M421I-ERα^LBD^ and Y537S-ERα^LBD^) is screened with E2, EE2, 4-NP and TAM ligands. 

The results show that the αβ/I peptide is able to distinguish between the ERα^LBD^s bound to strong agonists, (E2, EE2), weak agonist (4-NP) and antagonist (TAM) molecules. 

The assay which combines of the use of conformation-sensitive peptide and an array of ERα mutants with different affinities toward EDC compounds is a very promising tool for detecting the presence of/and potential estrogenic activity of chemicals through their binding to the ERα^LBD^. In addition, the regeneration capability of the platform and its adaptability to a portable SPR device make this assay promising for screening of EDCs in field.

## Supplementary Materials

The following are available online at www.mdpi.com/2079-6374/8/1/1/s1.
