# Supplementary material for: Label-Free Biosensor Detection of Endocrine Disrupting Compounds Using Engineered Estrogen Receptors
Source: Biosensors (Basel). 2017 Dec 22;8(1):1. doi: 10.3390/bios8010001 (PMC5872049; doi:10.3390/bios8010001)
Supplement: Supplementary file 1 [file biosensors-08-00001-s001.pdf]

Supplementary Material

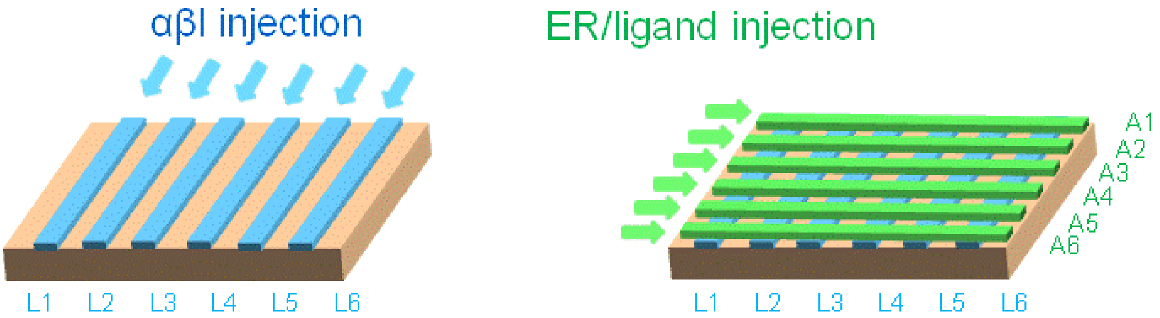

Fig 1S. Layout of the NLC chip surface, showing the vertical and horizontal direction for the injection of the αβ/I peptide and ER/ligand, respectively.

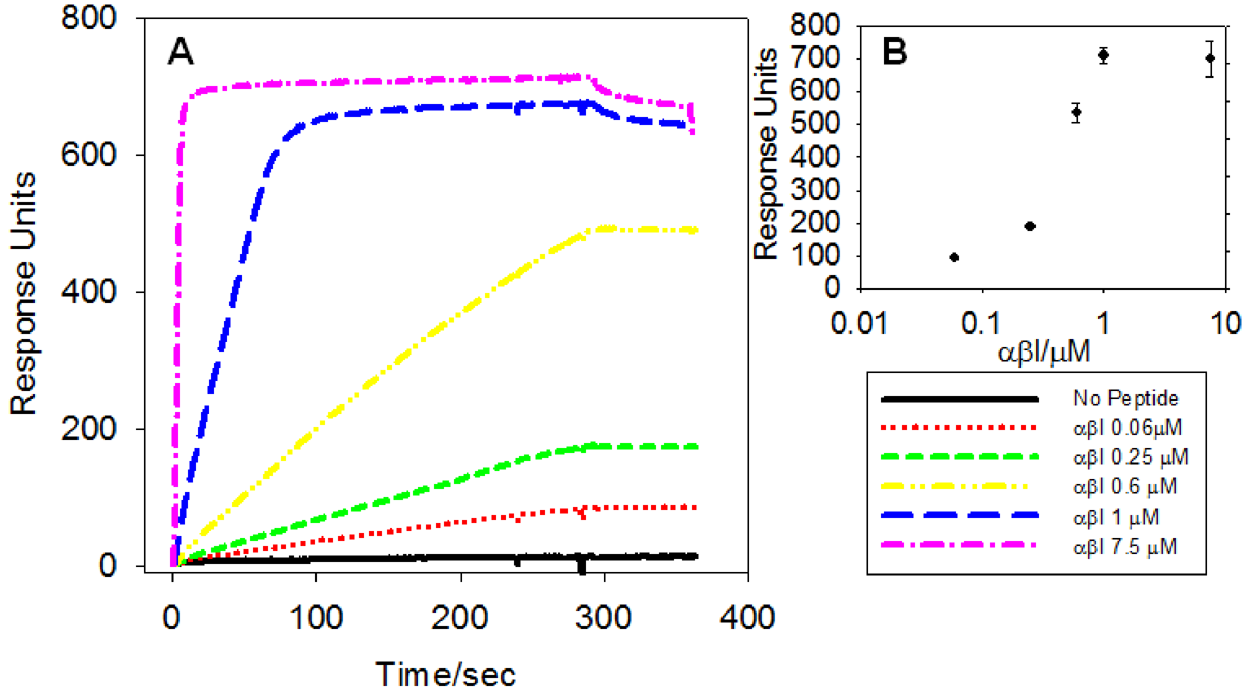

Fig 2S. **(A)** SPR sensorgrams showing the immobilization of αβ/I peptide at five different concentrations (0.06, 0.25, 0.6, 1.0 and 7.5 μM). One channel is the solvent reference channel and

it is subtracted to all signals, black continuous line. (**B)** Response unit values are plotted versus peptide concentration.

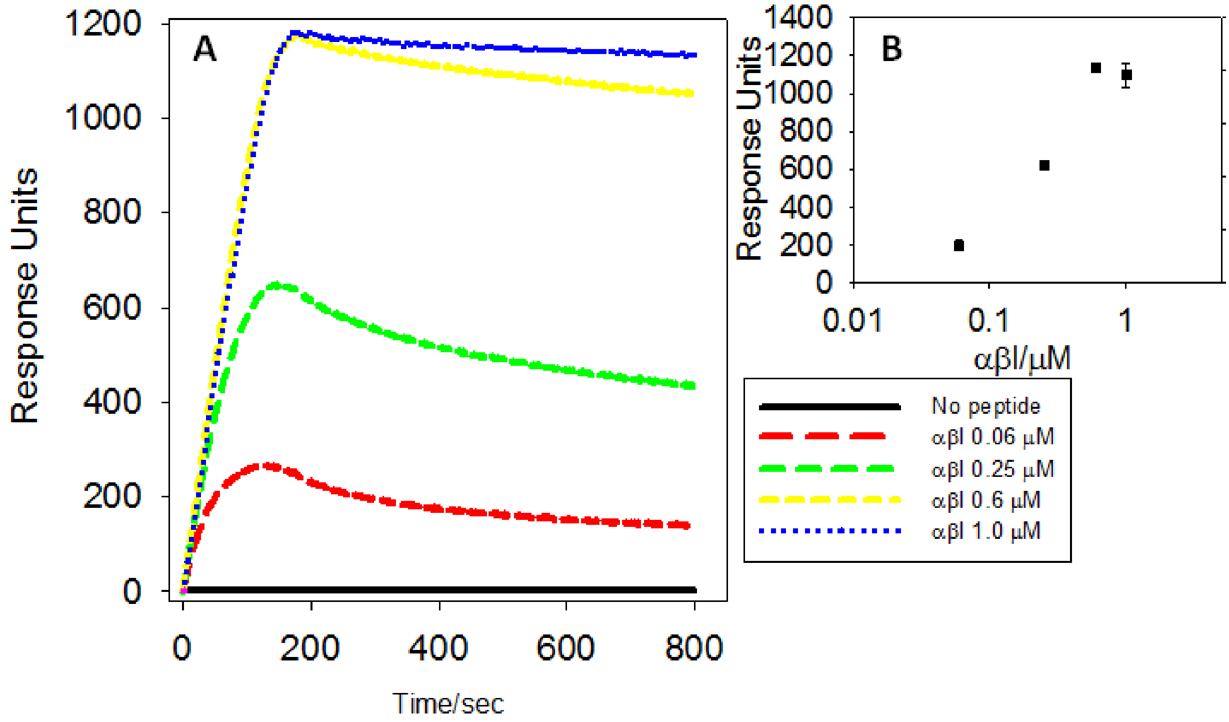

Fig 3S. (A) SPR sensorgrams showing the binding of wt-ERα^{LBD}-E2 complex (wt-ERα^{LBD} 134 nM and E2 1.5 μM) to four different concentrations of immobilized αβ/I peptide (0.06, 0.25, 0.6, and 1.0 μM). One channel without immobilized peptide is always used as a reference and subtracted to the other channels to take in account the unspecific binding of wt-ERα^{LBD}-E2 to the sensor surface, black continuous line. (**B)** Response unit values (recorded 60 sec after the end of injection) are plotted versus peptide concentration.

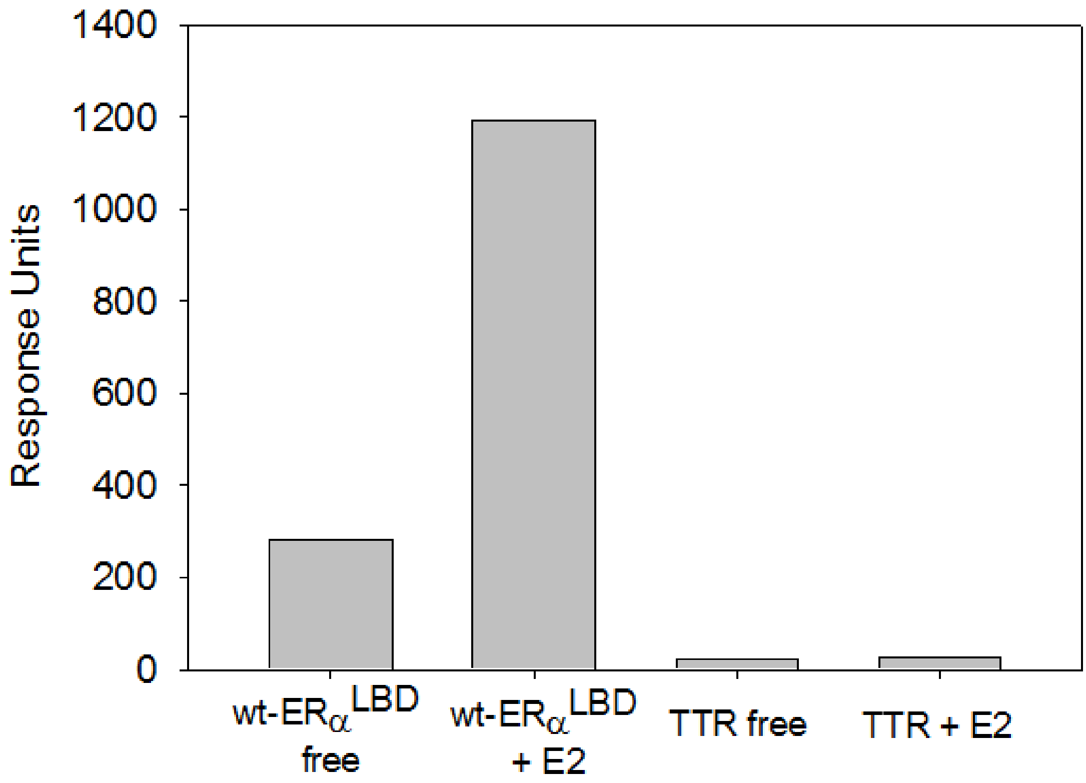

Fig 4S. Comparative SPR response of wt-ERα^{LBD} free, wt-ERα^{LBD} -E2 complex, TTR free and TTR-E2 complex. The SPR response is collected 60 sec after the end of injection. The data show that αβ/I peptides specifically recognize the active conformation of wt-ERα^{LBD} free and wt-ERα^{LBD} -E2 complex whereas no significant SPR signal is recorded for TTR and TTR-E2 complex.

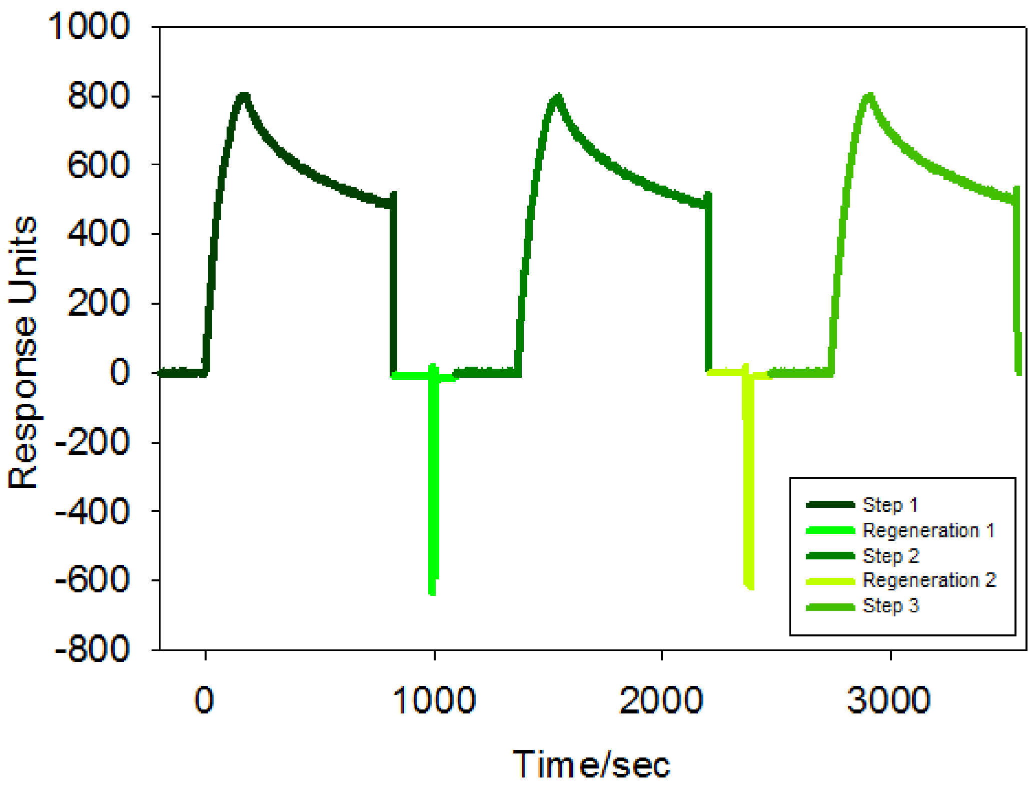

Fig 5S. SPR sensorgrams of 134nM wt-ERα^{LBD} incubated with 10 nM E2. The binding steps (Step 1, 2 ,3) and H_3PO_4 regenerations of the surface (Regeneration 1 and 2) are shown. Reproducibility of SPR response after regeneration is monitored checking the sensorgrams for three consecutive cycles.

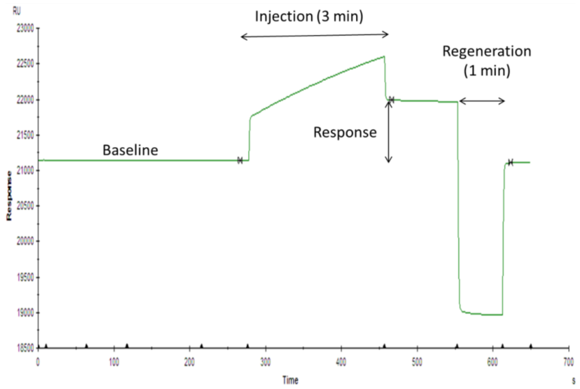

Fig 6S. Unreferenced sensorgram showing the baseline, the binding of wt-ERα^{LBD}-E2 to αβ/I amine peptide coated surface, the response obtained after the injection and the response during and after the regeneration. The total run time of one complete cycle is 11 min.

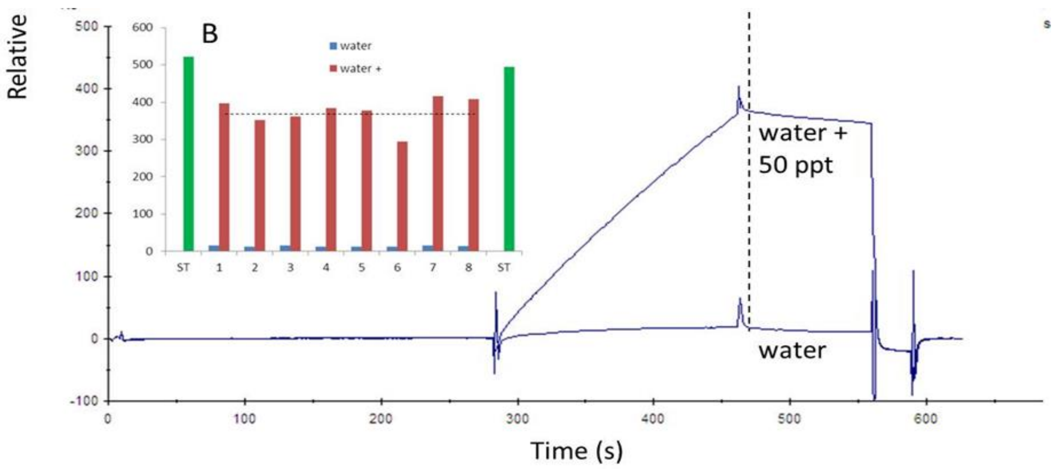

Fig 7S. Referenced sensorgrams obtained with a fish farm water sample from the UK and the same sample spiked with 50 ppt of E2 after SPE in which 50 mL of water was concentrated to 50 µL of methanol to which 200 µL of water was added and 80 µL was mixed with 80 µL of the wt-ERα^{LBD} and 90 µL was injected during 3 min. In total, 8 different real water samples were analysed with and without the addition of E2 (50 ppt) and the responses (insert) were compared with the responses (green) of standard solutions of E2 (10 ng/mL) corresponding with a 100% recovery.

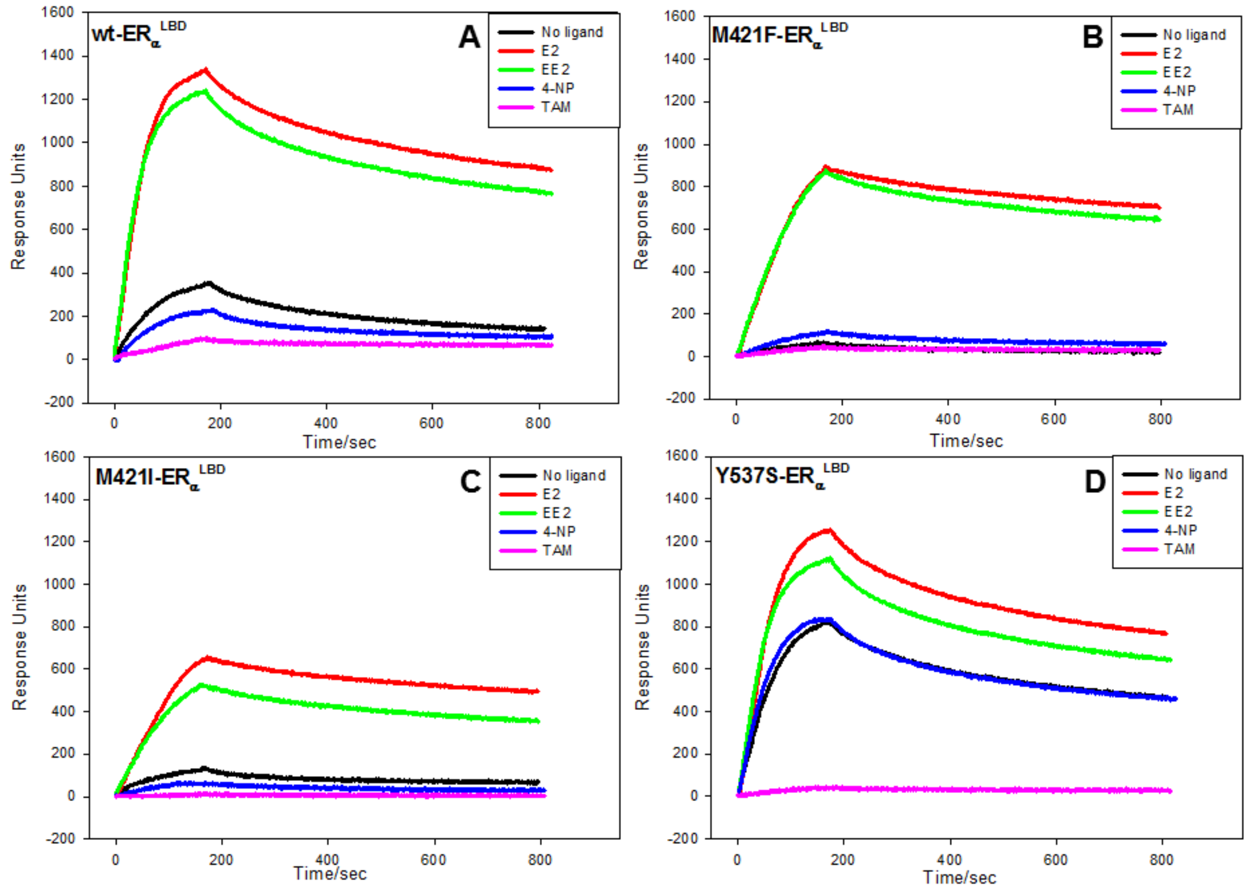

Fig 8S. SPR sensorgrams of ERα^{LBD}-ligands complexes onto αβ/I peptide. The concentration of ERα^{LBD} and ligands were 134 nM and 1500 nM, respectively. **(A)** wt-ERα^{LBD} **(B)** M421F-ERα^{LBD} **(C)** M421I-ERα^{LBD} **(D)** Y537S-ERα^{LBD} were incubated with several ligands: E2 (red line), EE2 (green line), TAM (violet line) 4-NP (blue line), or no ligand (black line).

ERα^{LBD} wild type and mutants present different affinities towards the agonists E2, EE2 and 4-NP [31] generating different of ERα^{LBD} active conformation i.e. different SPR signal responses (red, green and blue lines in Fig 8S).

Y537S-ERα^{LBD} complexes formed with strong agonistsE2 and EE2 generate a slightly signal larger than the ligand free one as a result of the natural active conformation of this receptor (Fig 8S D, red and green lines). The interactions of wt-ERα^{LBD}, M421F-ERα^{LBD} and M421I-ERα^{LBD} complexes formed with E2 and EE2 ligands lead to a much larger SPR response in comparison to ligand free

$ER\alpha^{LBD}$, as a results of the clear conformational change from steady (ligand free) to active (Fig 8S A, B, C red and green lines).

The SPR signal resulting from $ER\alpha^{LBD}$ - 4-NP binding is more difficult to interpret. The interaction of the different complexes with αβ/I peptide results in only a slight increase of the SPR response for M421F-$ER\alpha^{LBD}$ receptors and a decrease for the other receptors (wt-$ER\alpha^{LBD}$ and M421I-$ER\alpha^{LBD}$). Nevertheless, the difference in SPR response between $ER\alpha^{LBD}$-4NP and ligand free $ER\alpha^{LBD}$ is not very significant, probably due to the low affinity of this compound for used receptors. Consequently, it is difficult to reliably define a clear trend of the net change of SPR responses.

On the opposite, the interaction of $ER\alpha^{LBD}$ with the antagonist compound TAM, leads to a clear antagonist bound conformation (different from the active one), which is not recognized by αβ/I peptide, resulting in a very low SPR signal response (Fig 8S, magenta line) as compared to ligand free ones.
